# Predicting discharge location of hip fracture patients; the new discharge of hip fracture patients score

**DOI:** 10.1007/s00264-012-1526-5

**Published:** 2012-03-23

**Authors:** Anne Jochem Hendrik Vochteloo, Wim Eduard Tuinebreijer, Andrea Britta Maier, Rob Gerardus Henricus Hubertus Nelissen, Rolf Melchior Bloem, Peter Pilot

**Affiliations:** 1 Department of Orthopaedic Surgery, Leiden University Medical Centre, P.O. Box 9600, 2300 RC Leiden, The Netherlands; 2 Department of Orthopaedic Surgery, Reinier de Graaf Group, Delft, The Netherlands; 3 Department of Surgery-Traumatology, Erasmus MC, University Medical Centre Rotterdam, Rotterdam, The Netherlands; 4 Department of Gerontology and Geriatrics, Leiden University Medical Centre, Leiden, The Netherlands

## Abstract

**Purpose:**

This paper reports on the development and validity of a new instrument, called the discharge of hip fracture patients score (DHP), that predicts at admission the discharge location in patients living in their own home prior to hip fracture surgery.

**Methods:**

A total of 310 patients aged 50 years and above were included. Risk factors for discharge to an alternative location (DAL) were analysed with a multivariable regression analysis taking the admission variables into account with different weights based on the estimates. The score ranged from 0–100 points. The cut-off point for DAL was calculated using a ROC analysis. Reliability of the DHP was evaluated.

**Results:**

Risk factors for DAL were higher age, female gender, dementia, absence of a partner and a limited level of mobility. The cut-off point was set at 30 points, with a sensitivity of 83.8%, a specificity of 64.7% and positive predictive value of 79.2%.

**Conclusion:**

The DHP is a valid, simple and short instrument to be used at admission to predict discharge location of hip fracture patients.

## Introduction

The number of hip fracture patients is growing. It has been estimated that the total number of hip fracture patients aged 50 years and older will be around 6.3 million by 2050 worldwide [1, 2].

Traditionally, the focus of research on hip fracture patients has focussed on technical aspects, morbidity and mortality. However, in the last two decades social morbidity due to a more limited level of activities of daily living, loss of independence and a sudden change in place of residence has increasingly become the subject of research. Furthermore, costs of caring for this fragile population are rising [3, 4].

Discharge to an alternative location (DAL) or the necessity to arrange additional postoperative care at home for those that can go home directly after discharge can contribute to a longer stay in hospital and thus create additional costs [3, 4]. Early planning of the date of discharge and the type of discharge location can reduce these costs [4–6]. An instrument that predicts the discharge location at the time of admission would therefore be of great importance, not only for the liaison service but also for patients and their family. Although there are some publications about risk factors for DAL [7–11], few discharge prediction scores for hip fracture patients have been published [12–14]. These scores are of limited value in current daily practice as they are either relatively old, time consuming to fill out or not applicable at admission.

In this paper we offer a new instrument, the discharge of hip fracture patients score (DHP), that predicts on admission, the discharge location in patients living in their own home prior to admission for a hip fracture.

## Methods

### Patients

This is an analysis of a series of 498 consecutive hip fracture patients aged 50 years and older admitted to a 450-bed teaching hospital (Delft, The Netherlands) between January 2008 and December 2009. Patients with a fracture due to a high-energy trauma or with a pathological fracture were not included in this cohort. Only patients living in their own home prior to admission (*n* = 336) were included. Patients who were treated conservatively (*n* = 8), those with incomplete data (*n* = 11) and those who died during hospital stay (*n* = 7) were excluded from this group. Thus, 310 patients with complete data were analysed.

### Data collection

Uniform collection and recording of data of all patients was achieved by routine evaluation at admission, according to the standardised care pathway for hip fracture patients.

Demographic data collected were age, gender, presence of a partner and discharge location.

Characteristics obtained during hospital stay were American Society of Anesthesiologists (ASA) Physical Status classification, presence of dementia based upon history taking from patients, families and carers, presence of anaemia on admission based on the criteria of the World Health Organisation (haemoglobin level below 7.5 mmol/L [12 g/dL] in women and below 8.1 mmol/L [13 g/dL] in men), level of mobility and activities of daily living, type of fracture (intra- or extracapsular hip fracture), type of fracture treatment (osteosynthesis or arthroplasty), type of anaesthesia (general or spinal), diagnosis of dementia based on criteria of the DSM IV and length of stay (LOS) [15, 16].

### Pre-fracture level of mobility and activities of daily living

The level of mobility was divided into four main categories: mobile without the use of an aid in- and outdoors, mobile in- and outdoors with the use of an aid in- and/or outdoors, only mobile indoors (regardless of the use of an aid) and the last group was immobile both in- and outdoors. A cane, crutch(es) or walker were all considered an aid, patients in a wheelchair were considered to be immobile.

The Groningen activity restriction score (GARS) is a functional activities of daily living (ADL) score [16]. A summed score for basic ADL was calculated ranging from 18 (indicating ability to perform all activities without assistance or undue effort) to 72 (indicating disability). A higher GARS score therefore represents a lower level of ADL.

### Statistical analysis

Demographic continuous data are presented as means, with standard deviations (SD). Categorical data are presented as the number of subjects in the category, along with the percentages. A multivariable logistic regression analysis was used for analysis of patients that were discharged from hospital to an alternative location. As we wanted an instrument that predicted discharge to an alternative location (DAL) at admission, we used risk factors in the multivariable analysis that were known at admission: age, gender, presence of a partner, perioperative risk (ASA classification I/II or III/IV), presence of dementia, anaemia at admission, pre-fracture level of mobility (using the three categories of mobility and the GARS) and type of fracture (intra- or extracapsular hip fracture).

Patients classified ASA I or II and III or IV were combined into two groups, as the separate numbers of patients classified ASA I (*n* = 41) and ASA IV (*n* = 9) were too small to be analysed separately. Age was categorised into three groups: 50–64.9 years old, 65–79.9 years old and 80 years old and above.

For the multivariable logistic regression analysis, immobile patients (*n* = 3) were excluded due to small numbers.

The likelihood ratio backward test was used to find the best-fit model by selecting the variables one by one. The probability for entry was set at 0.05, and the probability for removal at 0.10. P-values lower than 0.05 were considered statistically significant.

### Discharge of hip fracture patients score (DHP)

All significant risk factors from the multivariable logistic regression analysis were used in the model as score items. Each item was assigned a weighing factor based on the beta-coefficient, in such a way that the score added up to a maximum of 100 points. As the subcategory “immobile” was excluded from this analysis, this item was assigned the same weighing factor as the “only mobile indoors” category.

### Clinical reliability and validity

A receiver operating characteristics curve (ROC) was created by plotting the sensitivity (true positive rate) versus the 1-specificity (false positive rate). The actual area under the curve measures the ability of the instrument to classify correctly the patients with and without a high risk for not being discharged to their own home, therewith identifying the best cut-off point.

The percentages of scores below 10 and above 90 points were calculated to assess floor and ceiling effects.

Spearman’s correlation coefficient was calculated between items of the DHP and DAL to determine the convergent validity. All data were analyzed in IBM SPSS Statistics 19.0 (SPSS Inc., Chicago, USA).

## Results

### Patients

Table 1 shows clinical characteristics of the cohort, stratified by discharge location. The mean (SD) age of all patients was 78.5 (10.5) years, and 67.1% were female. One hundred and nineteen (38.3%) patients were directly discharged from hospital to their own home. These patients were younger, less often female and in better general medical and social condition compared to those who were discharged to an alternative location. Furthermore, they had a shorter LOS.

**Table 1:** Clinical characteristics of the whole cohort and stratified by discharge location

Characteristics	Cohort *n *= 310	Discharge location		*P*-value^a^
		Own home *n *= 119	Alternative location *n *= 191	
Age category				<0.001
50–64 years old	45 (14.5)	36 (30.3)	9 (4.7)	
65–79 years old	107 (34.5)	54 (45.4)	53 (27.7)	
≥80 years old	158 (51.0)	29 (24.4)	129 (67.5)	
Female gender	208 (67.1)	63 (52.9)	145 (75.9)	<0.001
Dementia	29 (9.4)	28 (14.7)	1 (0.8)	<0.001
Partner at admission	174 (56.1)	44 (37.0)	130 (68.1)	<0.001
ASA classification III/IV	78 (25.2)	20 (16.8)	58 (30.4)	0.007
Anaemia at admission	100 (32.3)	28 (23.5)	72 (37.7)	0.009
Pre-fracture mobility				<0.001
Without an aid	164 (52.9)	92 (77.3)	72 (37.7)	
With an aid	127 (41.0)	26 (21.8)	101 (52.9)	
Only mobile indoors	16 (5.2)	0 (0.0)	16 (8.4)	
Immobile	3 (1.0)	1 (0.8)	2 (1.0)	
Mean GARS (SD)	32.8 (14.7)	24.8 (11.2)	37.8 (14.4)	<0.001
Intracapsular hip fracture	192 (61.9)	87 (73.1)	105 (55.0)	0.001
Osteosynthesis	195 (62.9)	88 (73.9)	107 (56.0)	0.001
Spinal anaesthesia	297 (95.8)	116 (97.5)	181 (94.8)	0.383
Mean LOS, days (SD)	12.3 (9.2)	8.6 (6.5)	14.6 (9.8)	<0.001

*LOS* length of stay, *GARS* Groningen activity restriction score, *ASA* American Society of Anesthesiologists Physical Status classification

Values are given as number (percentage) if not stated otherwise

^a^ Bivariate analysis

### Discharge of hip fracture patients score (DHP)

Based on outcome of the multivariable logistic regression analysis (Table 2), the model for the DHP (Table 3) was developed. Age (categorised in three groups), female gender, dementia, absence of a partner and a more limited level of mobility were used as score items. The weighing factor was calculated using the beta coefficient, demonstrated in Table 2.

**Table 2:** Risk factors identified for discharge to an alternative location, as identified with multivariable logistic regression analysis

Risk factors	Beta coefficient	OR	95 % CI	*P*-value
Age category^a^				
65-79.9 years	1.32	3.76	1.48–9.55	0.005
≥80 years	2.28	9.78	3.68–25.98	<0.001
Female gender	0.79	2.20	1.21–4.02	0.010
Dementia	2.30	9.98	1.23–80.85	0.031
Absence of a partner	0.87	2.39	1.33–4.29	0.004
Mobility category^b^				
Mobile with an aid	0.848	2.33	1.25–4.35	0.008
Only mobile indoors	3.58	35.9	3.68–350.1	0.002

*OR* odds ratio, *CI* confidence interval

^a^40–64.9 years (for age category)

^b^mobile without an aid (for mobility category)

**Table 3:** Discharge of hip fracture patients score

Predisposing risk factors for discharge to an alternative location	Points
Age category	
50–64.9 years	0
65–79.9 years	10
≥ 80 years	20
Female gender	10
Dementia	20
Absence of a partner	10
Mobility at admission	
Mobile in- and outside without an aid	0
Mobile in- and outside with an aid for either one or both	10
Only mobile indoors	40
Immobile	40
Total score	

### Clinical reliability and validity

**Fig. 1:**
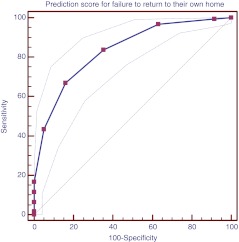
Receiver operating characteristic (ROC) curve of the prediction score with 95% confidence intervals. The diagonal indicates results no better than by chance

The ROC curve of the DHP is demonstrated in Fig. 1, where the area under the curve was 0.84 (95% CI 0.79–0.88). The best cut-off point for balancing sensitivity and specificity was 30 points. Sensitivity and specificity of the DHP at this cut-off point were 83.8% and 64.7%; the positive predictive value was 79.2%. The predictive power for two cut-off points (≥30 and ≥40 points) is shown in Table 4. The likelihood ratio of a DHP ≥30 was 2.37, which means that the probability of a score of ≥30 points being associated with DAL is 2.4 times higher compared to the probability that the patient is discharged to his or her own home.

**Table 4:** Results of the validity analysis of the prediction model for failure to return to own home at discharge

Measurement	≥30 points		≥40 points	
	Value	95 % CI	Value	95 % CI
Sensitivity %, (CI)	83.8	77.8–88.7	67.0	59.9–73.6
Specificity %, (CI)	64.7	55.4–73.2	84.0	76.2–90.1
Positive predictivity %, (CI)	79.2	72.8–84.4	87.1	80.3–91.8
Negative predictivity %, (CI)	71.3	61.7–79.4	61.3	53.4–68.8
Likelihood ratio positive (sens/1-spec)	2.37	2.0–2.7	4.20	3.7–4.8
Likelihood ratio negative (1-sens/spec)	0.25	0.20–0.40	0.39	0.20–0.60
Correlation with discharge to alternative location	0.50	n/a	0.50	n/a

*CI* confidence interval

Table 5 shows correlations, expressed with Spearman correlation coefficient, between DAL and the items of the DHP and the correlation between the items themselves. All items had a significant correlation with DAL, indicating good convergent validity.

**Table 5:** Spearman’s correlation coefficient between discharge to an alternative location and the items of the discharge in hip fracture patients score and between the items themselves

Measurement	DAL	Age categories	Female gender	Dementia	Absence of a partner
Age categories	0.459^b^				
Female gender	0.238^b^	0.139^a^			
Dementia	0.231^b^	0.206^b^	0.083		
Absence of a partner	0.305^b^	0.316^b^	0.261^b^	−0.005	
Mobility categories	0.392^b^	0.385^b^	0.157^b^	0.171^b^	0.155^b^

*DAL* discharge to an alternative location

^a^ Correlation is significant at the 0.05 level (2-tailed)

^b ^Correlation is significant at the 0.01 level (2-tailed)

The DHP scores ranged from zero to 90 points. The score was 10 or less points in 39 (16.1%) patients, thereby the floor effect was small. A DHP score of 90 points or more was present in only three (1.0%) patients, thus no ceiling effect was present.

## Discussion

A new prediction score for the discharge location of hip fracture patients living in their own home prior to admission was developed called the discharge of hip fracture patients score (DHP). This score was established using significant risk factors from multivariable regression analysis for failure to return to their own home: higher age, female gender, dementia, absence of a partner and a lower level of mobility. The score was easy to use due to readily obtainable data at admission and it had a good sensitivity and acceptable specificity.

The DHP can be used to facilitate the work of the liaison officers to make early arrangements for discharge, i.e. finding an appropriate alternative location or provide extra facilities (e.g. a walker or Zimmer frame, toilet seat risers, domestic help) needed in the first period after discharge. This can reduce LOS substantially [4–6].

### Patients

Mean age, the male to female ratio and fracture distribution of our series were comparable to a large cohort study of 3,683 hip fracture patients [17]. Another large series (3,240 patients) of Deakin et al. found a comparable mean age and percentage of patients living in their own home prior to admission [8]. The cohort we analysed was therefore representative for a general hip fracture population in Europe.

### Risk factors

We identified higher age, female gender, dementia, absence of a partner and a lower level of mobility as risk factors for DAL. Deakin et al. published the largest series on risk factors for DAL of hip fracture patients [8]. In that study, pre-injury level of dependence, higher age, male gender and injury sustained in hospital were identified as main risk factors for DAL. Other smaller studies identified age, number or type of comorbidities or a poor general health status, dementia, absence of a partner, a trochanteric fracture, a more limited level of mobility and ADL or the expectations of the nursing staff as predictors for DAL [7, 9–14]. In our study, poor general health (i.e. a higher ASA score) was identified as a predictor in the bivariate analysis, but lost its significance in the multivariate analysis.

### Other models

Currently, few prediction scores for discharge location after admission following a hip fracture are available [12–14]. However, these scores have their drawbacks. One model is more than 30 years old, thus data cannot be extrapolated to current day and practice [13]. Furthermore, all these models are based on small patient series (63–108 patients) [12–14]. Finally, one score used items that were not yet known at admission, i.e. the level of ADL and mobility two weeks postoperatively [14]. Nevertheless, these three models did use similar risk factors as we used in our score [12–14]. Only female gender was identified as an additional item compared to previous models, but with a modest impact indicated by the lowest weighing factor.

When extrapolating the results of our study to different countries, one must be aware of various possible influencing factors. First, there are large differences between countries in type of housing and traditions for homes for elderly people [18]. Second, as for the location and timing of discharge from the hospital, large local, national and international differences exist between discharge directly to home or to temporary revalidation units. This is reflected in the very wide range (3–81%) of reported rates of discharge to home directly from hospital [7, 12–14, 19–23].

### Clinical reliability and validity

The sensitivity of the DHP was good, the specificity moderate, which is good as is it of more importance to have a high number of true-positives (i.e. those patients that were labelled DAL, really had a DAL) in a prediction model. Because of the small floor effect, it is not possible to further differentiate within patients with low scores. However, this is not a real issue as the model is developed for only two options: discharge to their own home or to an alternative location.

### Limitations

Although this study describes a simple, valuable prediction model for discharge location in a well-sized cohort of hip fracture patients, some limitations remain. The first limitation was absence of more detailed data of cognitive function. Another limitation is the fact that the mobility category “immobile” and the category “only mobile indoors” were combined to one group due to the small number of patients who were immobile (*n* = 3). Finally, this is a model for the Dutch social situation and might not be applicable to other countries. However, the score items we have used were very comparable to those in previous models from other countries (United Kingdom, Sweden, United States) [12–14].

## Conclusions

In this paper we presented a useful new score that predicts discharge location at admission in hip fracture patients living in their own home prior to admission. In the near future, we will validate this model and test its inter-observer reliability in another Dutch and a Swedish hospital to prove the reliability of the model in different hospitals and countries, therewith allowing better (international) comparison.

## Abbreviations

DHP: Discharge of hip fracture patients score
DAL: Discharge to an alternative location
ASA: American Society of Anesthesiologists
LOS: Length of stay
GARS: Groningen activity restriction score
ADL: Activities of daily living
SD: Standard deviation
OR: Odds ratio
CI: Confidence interval
ROC: Receiver operating characteristics curve
